# Endocrine therapies and mortality risk in postmenopausal women with breast cancer: benchmarking an observational analysis against a randomized trial

**DOI:** 10.1093/aje/kwaf183

**Published:** 2025-08-21

**Authors:** Ali Al-kassab-Córdova, Anna B C Humphreys, Camila Olarte Parra, Maria Feychting, Anthony A Matthews

**Affiliations:** Unit of Epidemiology, Institute of Environmental Medicine, Karolinska Institutet, Stockholm, Sweden; Centro de Excelencia en Investigaciones Económicas y Sociales en Salud, Universidad San Ignacio de Loyola, Lima, Peru; Unit of Epidemiology, Institute of Environmental Medicine, Karolinska Institutet, Stockholm, Sweden; Unit of Epidemiology, Institute of Environmental Medicine, Karolinska Institutet, Stockholm, Sweden; Unit of Epidemiology, Institute of Environmental Medicine, Karolinska Institutet, Stockholm, Sweden; Unit of Epidemiology, Institute of Environmental Medicine, Karolinska Institutet, Stockholm, Sweden

**Keywords:** benchmarking, observational studies, clinical trial, target trial emulation, breast cancer

## Abstract

Benchmarking an observational analysis against a randomized trial increases our confidence in the use of observational data for causal inference. The Breast International Group (BIG 1-98) randomized trial compared the effect of letrozole and tamoxifen on the risk of death in postmenopausal women with hormone receptor–positive breast cancer. We designed a target trial that aimed to ask the same question as the one asked in BIG 1-98 and emulated it in Swedish registry data. The primary results from our observational analysis showed an increased risk of death in those who initiated aromatase inhibitors compared with tamoxifen [5-year risk difference = 2.5% (95% CI, 0.2-4.6)], which was discordant to the results from BIG 1-98. However, estimates were more closely aligned when our observational analysis was restricted to nonusers of opioids or antidepressants [5-year risk difference = −0.9 (95% CI, −4.2 to 2.0)]. In conclusion, when benchmarking an observational analysis against a trial, alignment of eligibility criteria with the index trial is not always sufficient and further study population restrictions may be required to address unmeasured confounding.

## Introduction

Benchmarking an observational analysis against a randomized trial increases confidence in the use of observational data for causal inference.^1^^-^^3^ There is growing interest in benchmarking, with results from observational studies being compared to those from similar randomized trials across a range of clinical areas.^1^^,^^4^^-^^7^

Before benchmarking, the natural first step is to design the protocol of a target trial that both aligns with the protocol of the index trial of interest and can be emulated in the available observational data. If the available observational data does not include information on the same variables as collected and defined in the trial, it is not possible to align all components of the study design, that is, the index trial and the target trial will be different. The question then becomes: are these differences small enough that the index trial and target trial ask the same question? If not, then any comparison of results between the index trial and the observational emulation of the target trial becomes irrelevant. Even if they estimate similar results, the studies ask different questions, so the comparison holds no coherence.

Even with perfect alignment between the protocol of the index and target trials, confounding in the observational analysis can still be a major issue. Treatment is assigned under routine clinical practice in the observational data, so the observational analyses require detailed data on baseline confounders. Yet sometimes, the nuanced reasons individuals receive one treatment over another cannot be accurately captured in data; there remains unmeasured confounding.

Here, we designed a target trial that aims to address the same research question as one of the analyses conducted in the Breast International Group 1-98 (BIG 1-98) trial: what is the comparative risk of death between aromatase inhibitors and tamoxifen in postmenopausal women with estrogen or progesterone receptor–positive breast cancer?^8^ We then emulated this target trial using data from Swedish healthcare registries and benchmarked the results of the two studies.

## The index randomized trial: BIG 1-98

The BIG 1-98 (ClinicalTrials.gov ID: NCT00004205) was a multicountry double-blinded, phase III cluster-randomized clinical trial. One of the comparisons in BIG 1-98 was the effect of monotherapy with letrozole (an aromatase inhibitor) or tamoxifen as adjuvant endocrine therapy for postmenopausal women with hormone receptor–positive breast cancer on the risk of death. Of note, the treatments were cluster randomized at the treatment center level. The trial was conducted between March 1998 and May 2003 in 240 participating centers worldwide, enrolling 8010 participants, of whom 4922 received monotherapy with either letrozole or tamoxifen. For the analysis, hazard ratios (HRs) and their 95% confidence intervals (95% CI) were estimated via Cox proportional hazards model. Further details of the trial,^8^^,^^9^ including the baseline characteristics of the participants,^10^ are published elsewhere. A summary of the protocol is available in Table 1.

**Table 1:** Description of Breast International Group (BIG 1-98) randomized trial, target trial, and target trial emulation using the National Breast Cancer Registry.

**Protocol component**	**BIG 1-98 (Index randomized trial)**	**Target trial**	**Target trial emulation**
**Eligibility criteria**	– Postmenopausal women with operable breast cancer between March 1998 and May 2003 ER and/PgR ≥10 fmol/mg cutosol protein; or ≥10% of tumor cells positive by immunocytochemical evaluation Received adequate mastectomy, lumpectomy, or quadrantectomy Adequate hematological and renal function No distant metastases No bilateral breast cancer No previous malignancy within the previous 5 years except for adequately treated basal or squamous cell carcinoma No other nonmalignant systemic diseases Not known to be HIV positive Not receiving adjuvant chemotherapy at randomization No treatment with investigational drugs within 30 days Written informed consent	Same as index trial, apart from: – Women aged 40 years and older Within 9 months of primary surgery that resulted in clear margins No requirement for adequate hematological and renal function, and negative HIV status Study period between August 2009 and December 2015	Same as target trial, apart from: – No informed consent was required
**Treatment strategies**	*Two-arm monotherapy clinical trial* (1) Letrozole 2.5 mg daily for 5 years (2) Tamoxifen 20 mg daily for 5 years	*Two-arm target trial* (1) Initiation of aromatase inhibitor (letrozole, anastrozole, or exemestane) and continuation for the duration of follow-up. (2) Initiation of tamoxifen and continuation for the duration of follow-up. Under all strategies, therapy can be discontinued if/when a contraindication arises. The treating physician determines the treatment dose.	Assignment is defined as the date of the first dispensation of either treatment recorded in the Prescribed Drug Register. Discontinuation dates are calculated using the number of days of the prescription. Treatment was considered continuous if there was a gap of less than 60 days between successive dispensations.
**Treatment assignment**	Participants were randomly assigned, in a blinded manner, to a treatment strategy (by center).	Individuals randomly assigned to a strategy at baseline and are aware of the assigned strategy.	Individuals assigned to the strategy their data are compatible with, and assignment assumed to be randomized within levels of the baseline covariates (age, year of baseline, time from diagnosis, stage at diagnosis, T classification, N classification, grade, HER2 status, side, chemotherapy, radiotherapy, antibody treatment, cerebrovascular disease, diabetes, COPD, cardiovascular disease, anticoagulants, antidepressants, NSAIDs, opioids, hormone replacement therapy, marital status, education status, and employment status).^a^
**Outcome**	Death at 5 years	Same as index trial	Same as target trial
**Follow-up**	For 5 years, patients attended clinic visits every 6 months for follow-up to collect safety data and obtain a new supply of study medications. Patients were followed up lifelong for disease status, survival, and certain adverse events.	For each eligible individual, follow-up starts at treatment assignment (baseline) and ends at the outcome of interest, loss to follow-up (emigration), 5 years, or administrative end of follow-up (December 31, 2015), whichever happens first.	Same as target trial
**Causal contrasts**	Intention-to-treat effect	Intention-to-treat effect and per-protocol effect	The effect of assignment (analogue of the intention-to-treat effect) and per-protocol effects
**Statistical analysis**	Kaplan-Meier plots. Treatment differences were evaluated with log-rank test. HRs and 95% CI were estimated from a Cox proportional hazards model.	5-year risks estimated using pooled logistic regression model. Covariates unbalanced at baseline used to estimate inverse probability treatment weights. Per-protocol analysis is the same except individuals censored when they deviate from assigned treatment, and inverse probability censoring weights to adjust for baseline and time-varying variables. Nonparametric bootstrapping with 1000 samples to calculate 95% CI.	Same as target trial with baseline covariates included in estimation of inverse probability treatment weights. For the per-protocol effect, baseline and time-varying variables were included.

Abbreviations: ER, estrogen receptor; PgR, progesterone receptor; HR, hazard ratio; CI, confidence interval; COPD, chronic obstructive pulmonary disease; NSAIDS, nonsteroidal anti-inflammatory drugs.

a All ICD-10, NOMESCO, and ATC codes were used to define diagnosis, procedures, and treatments, respectively.

A total of 4922 consenting individuals were randomly assigned to receive either monotherapy with letrozole (2463 participants) or tamoxifen (2459 participants). In an intention-to-treat analysis, at a median follow-up time of 51 months, the 5-year risk of death was 9.2% and 9.9% for letrozole and tamoxifen groups, respectively; resulting in an HR of 0.91 (95% CI, 0.75-1.11).^10^

## The target trial

The target trial protocol was designed to be as similar as possible to the protocol of BIG 1-98, within the restrictions imposed by the available observational data (more detail on the data below). The protocol of the target trial is available in Table 1, and the main differences between the protocol of the BIG 1-98 trial and the target trial are discussed below.

Like BIG 1-98, the target trial would recruit postmenopausal women with estrogen or progesterone receptor–positive breast cancer. However, there are several differences in respect to the eligibility criteria. First, in the target trial, individuals from Sweden would be over 40 years of age and recruited between August 2009 and December 31, 2015. Second, women would be required to have received primary surgery that resulted in clear margins rather than only having operable breast cancer. Third, there would be no requirement for adequate hematological and renal function, and negative HIV status. All of these differences are a consequence of the restrictions imposed by data used to emulate the target trial.

The treatment strategies would be similar to the index trial. Eligible individuals would be assigned to any aromatase inhibitors (letrozole, anastrozole, or exemestane) or tamoxifen, instructed to continuously take the treatment for the duration of follow-up, and the dosage would be left to the discretion of the recruiting clinician. The target trial would randomly assign individuals to a treatment strategy. Women would also be encouraged to continue taking treatment unless they experience a contraindication (which although not explicitly stated in BIG 1-98, was likely what happened in practice). The outcome, death by 5 years, and length of follow-up would be the same as the index trial. In addition to estimating the intention-to-treat effect, the target trial would estimate the per protocol effect—that is, the effect under full adherence to the treatment strategies as specified in the protocol. While the index trial did not report a per-protocol estimate, adherence in the trial was very high. Consequently, the intention-to-treat estimate of the trial closely reflects the per-protocol effect of the target trial, allowing a fair comparison.

For the intention-to-treat analysis, absolute risks would be estimated parametrically using a smooth function of time, by fitting a pooled logistic regression model with an indicator for assigned strategy, time of follow-up (restricted cubic spline with knots at 6, 12, 24, and 36 months), and a product term between the assignment indicator and time of follow-up. The 5-year risk in each group would then be compared via risk differences and ratios. An estimate analogous to the HR from a Cox model would be obtained using the pooled logistic regression model without the assignment—time product term.^11^ Inverse probability (IP) weighting would be used to adjust for prognostic factors that are imbalanced at baseline (see Table 1), that is, each individual receives an IP weight whose denominator is the probability of being assigned to the individual’s treatment strategy conditional on the prognostic factors. These probabilities would be estimated via a logistic regression model.^12^ The per protocol analysis in the target trial would be similar to the intention-to-treat analysis except participants would be censored if and when they deviate from their assigned treatment. To adjust for the potential selection bias induced by this censoring, we need to adjust for prognostic factors that are associated with nonadherence. Given the available data and clinical judgment, we select the following postbaseline prognostic factors: cerebrovascular disease, diabetes, chronic obstructive pulmonary disease (COPD), cardiovascular disease, chronic kidney disease, diabetes drugs, anticoagulants, antidepressants, nonsteroidal anti-inflammatory drugs (NSAIDs), opioids, and hormone replacement therapy.

## The observational emulation of the target trial

The target trial was emulated using data from the Swedish National Quality Registry for Breast Cancer. The Breast Cancer registry was launched in 2008 to facilitate nationwide collection of data of all primary invasive and *in situ* breast cancer cases across Sweden.^13^ It comprises data on predefined diagnoses, therapeutic procedures, and death during follow-up. These data were linked to the National Cancer Register, the National Patient Register (ie, inpatient and outpatient patients),^14^ the National Cause of Death Register,^15^ the National Prescribed Drug Register,^16^ and the Longitudinal Integration Database for Health Insurance and Labor Market Studies (LISA).^17^

Each component of the target trial protocol above was emulated as closely as possible using these data, which is fully outlined in Table 1. As per all observational analyses, it was not possible to ask informed consent. Additionally, assignment to aromatase inhibitors or tamoxifen was operationalized as dispensation of either treatment at a pharmacy in Sweden, which is recorded in the Prescribed Drug Register.^16^ Discontinuations due to the development of a contraindication were not considered a deviation of the protocol. Other discontinuations, such as stopping for nonmedical reasons or switching drugs, were considered deviations from the assigned treatment strategy. Continuous use of either treatment was defined as not switching or a gap of less than 60 days between successive dispensations, and the length of each dispensation was calculated from the number of pills multiplied by the dose, divided by the defined daily dose.

Assignment to either aromatase inhibitors or tamoxifen (defined as first dispensation) was assumed to be random within levels of the baseline covariates: age, year of baseline, time from diagnosis, stage, T classification, N classification, grade, HER2 status, side, chemotherapy, radiotherapy, antibody treatment, cerebrovascular disease, diabetes, COPD, diabetic drugs, anticoagulants, antidepressants, NSAIDs, opioids, hormone replacement therapy, marital status, education status, and employment status (see Table S1 for full definition of all covariates).

Both the intention-to-treat and per-protocol analyses followed the target trial protocol and were additionally adjusted for all baseline covariates. For all covariates that had missing data, a missing data category was included. Subgroup analyses were conducted using the intention-to-treat approach within levels of: age (under and over 65 years old), administration of chemotherapy, and use of either opioids or antidepressants. For all analyses, nonparametric bootstrapping with 1000 samples was used to calculate 95% CI.

To assess the robustness and consistency of our estimates, the following sensitivity analyses were performed using the intention-to-treat approach: (1) a sensitivity analysis including baseline covariates in the pooled logistic regression models instead of IP weighting to compare different modeling approaches; and (2) a complete case analysis to assess the impact of missing data assumptions on the results.

Ethical approval was obtained from the Regional Ethical Review Board in Stockholm (2011/634-31/4, 2019-05224). Pseudonymized personal data was obtained and treated with confidentiality, ensuring the privacy and security of information.

## Results

**Figure 1:**
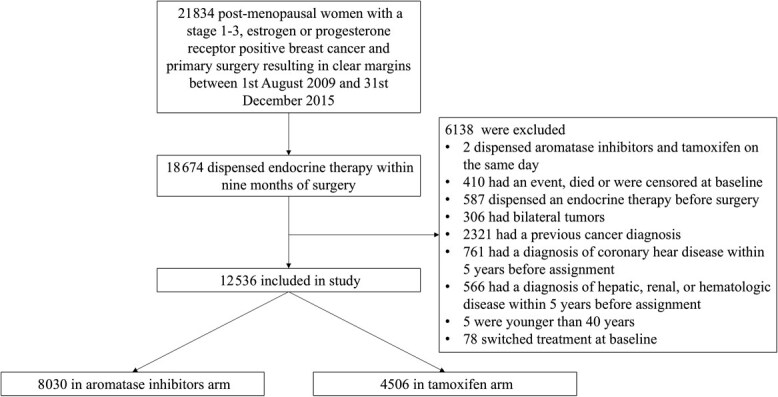
Flowchart of individuals eligible for an observational emulation of a target trial of aromatase inhibitors vs tamoxifen.

A total of 21 834 postmenopausal women with a stage 1-3 estrogen or progesterone receptor–positive breast cancer treated with primary surgery resulting in clear margins were identified between August 1, 2009 and December 31, 2015. After applying exclusion criteria, 12 536 individuals were included in the study, of whom 8030 received aromatase inhibitors and 4506 tamoxifen (flowchart for selection in Figure 1).

The median age at baseline (first dispensation) was 68 and 67 years in the aromatase inhibitor and tamoxifen groups, respectively, and most participants in both groups were diagnosed at stage 1, with T1 and N0 classifications. Those in the aromatase inhibitor group started treatment at a median of 102 days postdiagnosis (IQR 61.0-203.0), compared to 68 days (IQR 50.0-104.0) for the tamoxifen group. In addition, 30% of individuals in the aromatase inhibitors group received chemotherapy, compared with 6% in the tamoxifen group. Other baseline characteristics are presented in Table 2.

**Table 2:** Baseline characteristics of eligible individuals from an observational emulation of a target trial of aromatase inhibitors vs tamoxifen, Sweden, 2009-2015.

**Characteristics**	**Aromatase inhibitors (*n* = 8030)**	**Tamoxifen (*n* = 4506)**
	***n* (%)**	***n* (%)**
Age, years^a^	68.0 [62.0-75.0]	67.0 [61.0-73.0]
Year of baseline		
2009	628 (7.8)	611 (13.6)
2010	905 (11.3)	722 (16.0)
2011	1047 (13.0)	706 (15.7)
2012	1047 (13.0)	649 (14.4)
2013	1232 (15.3)	689 (15.3)
2014	1606 (20.0)	574 (12.7)
2015	1565 (19.5)	555 (12.3)
Time from diagnosis, days	102.0 [61.0-203.0]	68.0 [50.0-104.0]
Stage at diagnosis		
Stage 1	4021 (50.1)	3356 (74.5)
Stage 2	3695 (46.0)	1117 (24.8)
Stage 3	314 (3.9)	33 (0.7)
T classification		
T1	4422 (55.1)	3421 (75.9)
T2	3080 (38.4)	1006 (22.3)
T3/T4	528 (6.6)	79 (1.8)
N Classification		
N0	6725 (83.7)	4338 (96.3)
N1/N2/N3	1305 (16.3)	168 (3.7)
Grade		
Grade 1	1070 (13.3)	1302 (28.9)
Grade 2	4230 (52.7)	2726 (60.5)
Grade 3	2338 (29.1)	423 (9.4)
Missing	392 (4.9)	55 (1.2)
HER2 status		
Positive	977 (12.2)	138 (3.1)
Negative	6734 (83.9)	4101 (91.0)
Missing	319 (4.0)	267 (5.9)
Side		
Right	3826 (47.7)	2210 (49.0)
Left	4204 (52.3)	2296 (51.0)
Chemotherapy	2437 (30.3)	272 (6.0)
Radiotherapy	1921 (23.9)	866 (19.2)
Antibody treatment	677 (8.4)	52 (1.2)
Cerebrovascular disease	205 (2.6)	55 (1.2)
Diabetes	560 (7.0)	206 (4.6)
COPD	227 (2.8)	111 (2.5)
Cardiovascular disease	2948 (36.7)	1347 (29.9)
Diabetes drugs	577 (7.2)	217 (4.8)
Anticoagulants	2441 (30.4)	934 (20.7)
Antidepressants	1549 (19.3)	839 (18.6)
NSAIDs	3242 (40.4)	1839 (40.8)
Opioids	2552 (31.8)	1223 (27.1)
Hormone replacement therapy	2509 (31.2)	1521 (33.8)
Marital status		
Single	997 (12.4)	551 (12.2)
Married/cohabiting	4739 (59.0)	2673 (59.3)
Divorced/separated/widowed	2294 (28.6)	1282 (28.5)
Education status		
Pre-secondary education	2281 (28.4)	1201 (26.7)
High school education	3282 (40.9)	1854 (41.1)
Post-secondary education	2363 (29.4)	1408 (31.2)
Missing	104 (1.3)	43 (1.0)
Employment status		
Employed	4411 (54.9)	2611 (57.9)
Not employed	3598 (44.8)	1889 (41.9)
Missing	21 (0.3)	6 (0.1)

Abbreviations: COPD, chronic obstructive pulmonary disease; NSAIDS, nonsteroidal anti-inflammatory drugs. Only grade, HER2 status, education status, and employment status had missing data.

a Values are expressed as median (IQR).

### The effect of dispensation of aromatase inhibitors vs tamoxifen

**Figure 2:**
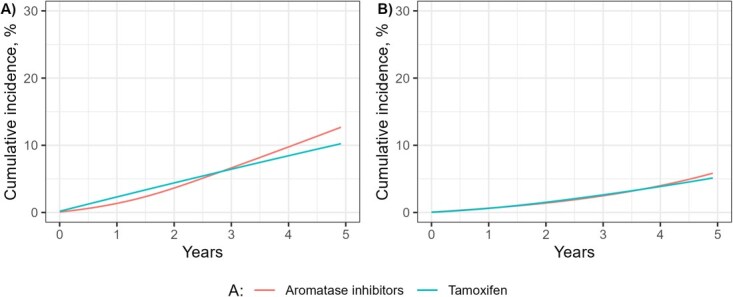
Adjusted cumulative incidence curves from an observational emulation of a target trial of aromatase inhibitors vs tamoxifen on death at 5 years. (A) Intention-to-treat analysis. (B) Per-protocol analysis. Time was modeled using splines at 6, 12, 24, and 36 months.

The estimated 5-year risks of death were 12.7% (95% CI, 11.5%-13.8%) with aromatase inhibitors and 10.2% (95% CI, 8.5%-12.1%) with tamoxifen, which results in a risk difference of 2.5% (95% CI, 0.2%-4.6%), a risk ratio of 1.24 (95% CI, 1.02-1.52), and an average HR by 5 years of 1.09 (95% CI, 0.89-1.34) (Table 3 and Figure 2).

**Table 3:** Absolute risks, RDs, RRs, and HRs from the BIG-98 randomized trial and an observational emulation of a target trial of aromatase inhibitors vs tamoxifen on death at 5 years.

	**Aromatase inhibitors**	**Tamoxifen**	**RD, %** **[95% CI]**	**RR** **[95% CI]**	**HR** **[95% CI]**
	**Risk, %** **[95% CI]**	**Risk, %** **[95% CI]**			
**BIG 1-98** ^**a**^					
ITT	9.2 [NR]	9.9 [NR]	−0.7 [NR]	0.93 [NR]	0.91 [0.75-1.11]
**Observational analysis**					
Effect of dispensation^b^	12.7 [11.5-13.8]	10.2 [8.5-12.1]	2.5 [0.2-4.6]	1.24 [1.02-1.52]	1.09 [0.89-1.34]
Per-protocol effect^c^	5.8 [4.8-7.1]	5.1 [3.8-6.6]	0.7 [−1.1 to 2.6]	1.14 [0.82-1.63]	1.01 [0.75-1.38]

Abbreviations: BIG 1-98, Breast International Group 1-98; HR, hazard ratio; ITT, intention-to-treat; NR, not reported; RD, risk difference; RR, risk ratio.

a Estimates at 5-year follow-up.^10^^,^^18^

b Adjusted at baseline for: age, year of baseline, time from diagnosis, type of surgery, stage at diagnosis, T classification, N classification, grade, side, chemotherapy, radiotherapy, antibody treatment, cerebrovascular disease, diabetes, COPD, cardiovascular disease, diabetes drugs, anticoagulants, antidepressants, NSAIDs, opioids, hormone replacement therapy, marital status, education status, and employment status.

c The model is adjusted for baseline and time-varying covariates.

### Per-protocol effect: the effect under full adherence to the treatment strategies

At 5 years, adherence was 58% in the aromatase inhibitor group and 57% in the tamoxifen group. After censoring at nonadherence to the assigned treatment strategy but before IP weighting, the median follow-up was 22 months in the aromatase inhibitor group and 27 months in the tamoxifen group. The estimated 5-year risks of death were 5.8% (95% CI, 4.8%-7.1%) with aromatase inhibitors and 5.1% (95% CI, 3.8%-6.6%) with tamoxifen, which results in a risk difference of 0.7% (95% CI, −1.1% to 2.6%), a risk ratio of 1.14 (95% CI, 0.82-1.63), and an average HR by 5 years of 1.01 (95% CI, 0.75-1.38) (Table 3 and Figure 2).

### Subgroup effects and sensitivity analyses

Table 4 shows full results for all subgroup and sensitivity analyses. There was little difference between the estimates within groups of age and chemotherapy administration. However, the comparative effect of aromatase inhibitors and tamoxifen on the risk of death by 5 years was heterogeneous within individuals with and without prior use of opioids or antidepressants, with the risk ratio by 5 years of 0.91 (95% CI, 0.68-1.25) and an average HR by 5 years of 0.79 (95% CI, 0.58-1.10) in nonusers of opioids or antidepressants. In contrast, among users of opioids or antidepressants, the 5-year risk ratio was 1.58 (95% CI, 1.22-2.09), with an average HR of 1.42 (95% CI, 1.08-1.92). Baseline characteristics and cumulative incidence curves from the analysis restricted to nonusers of opioids or antidepressants are in Table S2 and Figure S1, respectively. The subset of nonusers of opioids and antidepressants shows lower rates of comorbidities—including cardiovascular disease, COPD, diabetes, and cerebrovascular disease—as well as lower use of medications such as NSAIDs, anticoagulants, and hormone replacement therapy. Results for all sensitivity analyses were broadly similar to the main analysis (Table S3).

**Table 4:** Absolute risks, RDs, RRs, and HRs from the observational emulation of a target trial of aromatase inhibitors vs tamoxifen on death at 5 years, subgroup analyses.

	**Aromatase inhibitors**	**Tamoxifen**	**RD [95% CI]**	**RR [95% CI]**	**HR [95% CI]**
	**Risk, % [95% CI]**	**Risk, % [95% CI]**			
**Subgroup analyses**					
Age					
<65 years	6.3 [4.7-8.0]	4.9 [2.5-8.0]	1.4 [−2.1 to 4.3]	1.27 [0.73-2.62]	0.93 [0.53-1.93]
≥65 years	16.4 [14.8-18.0]	13.9 [10.8-17.2]	2.5 [−0.8 to 5.8]	1.18 [0.95-1.53]	1.07 [0.83-1.41]
Chemotherapy					
Administered	10.0 [7.8-12.3]	5.4 [1.6-9.9]	4.6 [−0.5 to 9.1]	1.86 [0.94-6.25]	1.38 [0.69-4.72]
Not administered	13.3 [12.1-14.7]	11.8 [10.0-14.2]	1.5 [−1.4 to 3.8]	1.13 [0.90-1.38]	1.02 [0.80-1.27]
Opioids or antidepressant					
Users	16.6 [14.7-18.6]	10.5 [8.1-13.3]	6.1 [2.9-9.2]	1.58 [1.22-2.09]	1.42 [1.08-1.92]
Nonusers	9.6 [8.4-11.0]	10.6 [7.9-13.8]	−0.9 [-4.2 to 2.0]	0.91 [0.68-1.25]	0.79 [0.58-1.10]

Abbreviations: BIG 1-98, Breast International Group 1-98; HR, hazard ratio; IPTW, inverse probability treatment weighting; ITT, intention-to-treat; NR, not reported; RD, risk difference; RR, risk ratio. Note: ITT analysis was conducted using IPTW of baseline covariates (age, year of baseline, time from diagnosis, type of surgery, stage at diagnosis, T classification, N classification, grade, side, chemotherapy, radiotherapy, antibody treatment, cerebrovascular disease, diabetes, COPD, cardiovascular disease, diabetes drugs, anticoagulants, antidepressants, NSAIDs, opioids, hormone replacement therapy, marital status, education status, and employment status).

## Discussion

We used observational data from Swedish registers to emulate a two-arm parallel target trial, which aimed to ask a similar question to one asked in the BIG 1-98 randomized trial. The primary results from our observational analysis, that is, the risk ratio at 5 years of the effect of dispensation that shows a harmful effect of aromatase inhibitors, are discordant from the results of similar analyses in the trial, which showed a protective effect of aromatase inhibitors. Other results, like the HR at 5 years, favor neither treatment and are in accordance with BIG 1-98. Yet, what is clear, is that none of the main results from the observational analysis show a protective effect of aromatase inhibitors compared with tamoxifen. However, current clinical guidelines recommend the use of aromatase inhibitors due to their proven effect in increasing survival rates, as demonstrated in a meta-analysis in which the BIG 1-98 was included alongside similar trials.^19^ Below we discuss potential explanations for the varying conclusions between the trial and observational analysis.

### Differences between the protocols of BIG 1-98 and the target trial

If the protocols of the trial and target trial do not align then the 2 studies target a different estimand and hence address different questions, so different conclusions could be expected. Although we tried to align the protocols as closely as possible, there were some differences, most of which were a consequence of restrictions of the observational data.

First, the eligibility criteria differed between the protocols. There were different definitions of the type of surgery required to be eligible (operable surgery in BIG 1-98; surgery resulting in clear margins in the target trial), no requirement for adequate hematological and renal function, or negative HIV status in the target trial, and different study periods. It could be argued that even though the eligibility criteria were slightly different, none of these varying eligibility criteria would result in populations that differed in respect to the distribution of effect modifiers. In which case, one would expect similar results. That said, we found no evidence as to whether advances in care, that is, new concomitant treatment regimens, in the decade between BIG 1-98 and our observational analysis facilitate a modification of the effect estimated reported in the original trial.

Second, there was a small difference in treatment strategy definition between the studies. In the BIG 1-98, letrozole was assigned to everyone in the aromatase inhibitor group, while our observational analysis included individuals that dispensed any aromatase inhibitor (letrozole, anastrozole, or exemestane). Letrozole was uniquely used in BIG 1-98 because the trial was funded by Novartis, who produce letrozole and market it as Femara. Nevertheless, there is no evidence to suggest any substantial differences in efficacy and safety between different aromatase inhibitors, so this should not contribute to any difference in results.^20^

Third, the primary analysis in both studies aimed to estimate the effect of assignment to a treatment strategy (with assignment given randomly in the trial and defined as a dispensation of treatment in the observational analysis) regardless of what happened after. Yet, in this scenario, study-specific levels of adherence to a treatment strategy can explain differences in results of analyses. There were considerable differences in adherence to treatment between the 2 studies: BIG 1-98 reported approximately 95% adherence whereas our observational study reported approximately 58% adherence. This is problematic if the characteristics of those that do not adhere also predict the outcome. A per-protocol effect—representing the effect under full adherence to the treatment strategies—should remain the same across studies, assuming there are no other differences. Although BIG 1-98 did not undertake a per protocol analysis, relative estimates from our per protocol analysis were more closely aligned with the effect of assignment in the trial (which had high adherence). The absolute risks, on the contrary, were substantially lower in the observational emulation, potentially due to differences in baseline risk profiles of those that adhered to the treatment strategies. There remained, however, considerable uncertainty due to low precision.

### Confounding in the observational analysis

As with all observational analyses, there is a risk of unmeasured or residual confounding. Interestingly, in the subgroup analysis of nonusers of either opioids or antidepressants, estimates of both the absolute risk of death in each group and the comparative difference in risk between the groups align much closer with the results of BIG 1-98 (5-year risk of death with aromatase inhibitors 9.2% in BIG 1-98 and 9.6% in nonusers of opioids or antidepressants in the observational analysis; 5-year risk of death with tamoxifen 9.9% in BIG 1-98 and 10.6% in nonusers of opioids or antidepressants in the observational analysis). The main comorbidities and characteristics accompanying an opioid or antidepressant use are also strong predictors of multimorbidity; and several studies have reported lower survival among cancer patients using opioids.^21–24^ Consistently, cancer patients with multimorbidity also experience higher mortality rates.^25^ According to our findings, nonusers of opioids or antidepressants have lower prevalence of chronic diseases and take fewer medications. Thus, opioids and antidepressants are not confounders in and of themselves. By excluding those given opioids and antidepressants we restrict to a less sick subset of the population by proxy, resulting in a population with a lower prevalence of strong outcome predictors. It is, therefore, conceivable that there is less confounding in the analysis restricted to nonusers of opioids and antidepressants, which can explain the more concordant results. Otherwise, resorting to the mere adjustment for these covariates may still lead to residual confounding as they might not fully capture all the variation in multimorbidity; as evidenced in the main analysis. This does, however, slightly change the question to one in which we are only interested in the comparative effect of aromatase inhibitors and tamoxifen in those without prior opioid or antidepressant use.

### Distribution of population characteristics between the studies

If there is an unequal distribution of characteristics between the populations in each study, and any of these characteristics are effect modifiers between any 2 studies, then results will be different even if both studies are bias-free.^6^ Those included in our observational analysis were, on average, older and less likely to receive chemotherapy than those in the trial. There is no substantial empirical evidence to suggest treatment effect heterogeneity by age or chemotherapy use, mainly because no trial is large enough to estimate these subgroup effects precisely (like our subgroup analyses), and no meta-analyses have addressed the comparative risk of death within these subgroups. But if age or chemotherapy do modify the comparative effect of aromatase inhibitors and tamoxifen, this will explain some of the differences in results between the trial and our observational analysis. It is also possible there are currently unknown treatment effect modifiers, such as other comorbidities, that were also overrepresented in our observational analysis compared with BIG 1-98. This is highly feasible given it is well known that trials generally underrecruit individuals with a high comorbidity burden.^26^ Nevertheless, if individual-level trial data are available, it becomes possible to conduct joint analyses of trial and observational data. Such analyses allow for adjusting differences in the distribution of effect modifiers between the index trial and the observational study, as well as for extending inferences from the trial to a broader target population—by modeling outcome and trial participation, among other analytic approaches. Yet to achieve this, strong identifiability assumptions must hold.^27^^,^^28^

In conclusion, we designed and emulated a target trial that aimed to ask the same question as one asked in BIG 1-98, and the primary conclusions differed between the 2 studies. It is likely this difference was mostly a consequence of confounding in our observational analysis, which we were able to overcome through restricting the population to one with fewer comorbidities. While restriction can improve internal validity and make benchmarking more reliable, it may come at the price of asking a different question; from the effect of treatment in the same population as the trial, to the effect of treatment in the population with a narrower range of characteristics. This trade-off highlights a key tension in benchmarking: reducing confounding through restriction may come at the cost of generalizability and of aligning precisely with the trial’s original target population.

## Supplementary Material

Web_Material_kwaf183

## Data Availability

Pseudonymized personal data were acquired from national Swedish Registry holders following ethical approval and a confidentiality assessment. The Swedish regulations on sensitive data state that this can be used only under agreement of legal requirements.
