# Supplementary material for: Endocrine therapies and mortality risk in postmenopausal women with breast cancer: benchmarking an observational analysis against a randomized trial
Source: Am J Epidemiol. 2025 Aug 21;194(12):3483–91. doi: 10.1093/aje/kwaf183 (PMC12671965; doi:10.1093/aje/kwaf183)

SUPPLEMENTARY MATERIAL

Endocrine therapies and mortality risk in postmenopausal women with breast cancer: benchmarking an observational analysis against a randomized trial

Ali Al-kassab-Córdova; Anna B. C. Humphreys; Camila Olarte Parra; Maria Feychting; Anthony A. Matthews

Table of contents

Table S1. Covariate definitions

Table S2. Baseline Characteristics of Eligible Individuals Non-users of Antidepressants and/or Opioids from an Observational Emulation of a Target Trial of Aromatase Inhibitors Versus Tamoxifen

Table S3. Absolute Risks, Risk Differences, Risk Ratios and Hazard Ratios from the Observational Emulation of a Target Trial of Aromatase Inhibitors versus Tamoxifen on Death at 5 years, Sensitivity Analyses

Figure S1. Cumulative Incidence Curve from an Intention-to-Treat Analysis Observational Emulation of a Target Trial of Aromatase Inhibitors versus Tamoxifen on Death at 5 Years, within Non-users of Opioids and/or Antidepressants

Table S1. Covariate definitions

Covariate	Register	Definition	Values	Form
Age	Population register	Age at baseline		Linear, quadratic, cubic
Year of baseline	Breast cancer register	Year of baseline	2008–2015	Categorical
Time from diagnosis	Breast cancer register	Days between diagnosis and baseline		Linear, quadratic
Stage	Breast cancer register	Stage of breast cancer at diagnosis	1 = Stage 1<br>2 = Stage 2<br>3 = Stage 3	Categorical
T classification	Breast cancer register	T classification of tumour - tumour size	1 = T1 tumor smaller or equal to 20 mm<br>2 = T2 tumor larger than 20 mm and smaller or equal to 50 mm<br>3 = T3 tumor larger than 50 mm<br>4 = T4 tumour<br>NA = Missing	Categorical
N classification	Breast cancer register	N classification of tumour - spread to lymph nodes	0 = N0 no regional lymph node metastases<br>1 = N1/N2/N3 Free lymph node metastasis(es) in axillary ipsilateral OR fixed lymph node metastasis(es) in axillary ipsilateral or intramammary without axillary metastases OR lymph node metastasis(es) in fossa scl / icl ipsilateral or intramammary in combination with axillary metastases<br>NA = Missing	Categorical
Grade	Breast cancer register	Grade of breast cancer at diagnosis	1 = Grade 1<br>2 = Grade 2<br>3 = Grade 3<br>NA = Missing	Categorical
HER2 status	Breast cancer register	HER2 status of breast cancer at diagnosis	1 = Positive<br>2 = Negative<br>NA = Missing	Indicator
Side	Breast cancer register	Breast in which cancer is located at diagnosis	1 = Right<br>2 = Left	Indicator
Chemotherapy	Breast cancer register	Adjuvant chemotherapy before baseline (anthracyclines, docetaxel, piclataxel, or other chemotherapies)	1 = Yes<br>0 = No	Indicator
Radiotherapy	Breast cancer register	Neo-adjuvant or adjuvant radiotherapy before baseline	1 = Yes<br>0 = No	Indicator
Antibody treatment	Breast cancer register	Neo-adjuvant or adjuvant antibody treatment before baseline (trastuzumab, pertuzumab, or other antibody treatments)	1 = Yes<br>0 = No	Indicator
Cerebrovascular disease	Patient register	Diagnosis of cerebrovascular disease up to 5 years before baseline in inpatient or outpatient register, using primary or other diagnoses (ICD-10: I60-I68)	1 = Yes	Indicator

Covariate	Register	Definition	Values	Form
			0 = No	
Diabetes	Patient register	Diagnosis of diabetes up to 5 years before baseline in inpatient or outpatient register, using primary or other diagnoses (ICD-10: E10-E14)	1 = Yes<br>0 = No	Indicator
COPD	Patient register	Diagnosis of COPD up to 5 years before baseline in inpatient or outpatient register, using primary or other diagnoses (ICD-10: J41-J44)	1 = Yes<br>0 = No	Indicator
Chronic kidney disease	Patient register	Diagnosis of chronic kidney disease up to 5 years before baseline in inpatient or outpatient register, using primary or other diagnoses (ICD-10: N18)	1 = Yes<br>0 = No	Indicator
Cardiovascular disease	Patient register	Diagnosis of cardiovascular disease up to 5 years before baseline in inpatient or outpatient register, using primary or other diagnoses (ICD-10: I chapter, I00–I99)	1 = Yes<br>0 = No	Indicator
Diabetes drugs	Prescribed drug register	Dispensation of diabetes drug up to 3 years before baseline (ATC: A10)	1 = Yes<br>0 = No	Indicator
Anticoagulants	Prescribed drug register	Dispensation of aticoagulants up to 3 years before baseline (ATC: B01)	1 = Yes<br>0 = No	Indicator
Antidepressants	Prescribed drug register	Dispensation of antidepressants up to 3 years before baseline (ATC: N06A)	1 = Yes<br>0 = No	Indicator
NSAIDs	Prescribed drug register	Dispensation of NSAIDs up to 3 years before baseline (ATC: M01A)	1 = Yes<br>0 = No	Indicator
Opioids	Prescribed drug register	Dispensation of opioids up to 3 years before baseline (ATC: A10)	1 = Yes<br>0 = No	Indicator
Hormone replacement therapy	Prescribed drug register	Dispensation of hormone replacement therapy up to 3 years before baseline (ATC: G03C, G03D, G03F, G03XC, G02BA03)	1 = Yes<br>0 = No	Indicator
Marital status	LISA	Latest marital status at end of year status prior to baseline	1 = Single<br>2 = Married/cohabiting<br>3 = Divorced/separated/widowed<br>NA = missing	Categorical
Education status	LISA	Latest education status at end of year status prior to baseline	1 = Pre-secondary education<br>2 = High school education<br>3 = Post-secondary education<br>NA = missing	Categorical
Employment status	LISA	Latest employment status at end of year status prior to baseline	1 = Employed<br>2 = Not employed<br>NA = missing	Categorical

Abbreviations: NSAIDs, Non-Steroidal Anti-Inflammatory Drugs; COPD, Chronic Pulmonary Obstructive Disease; LISA, Swedish Longitudinal Integrated Database for Health Insurance and Labour Market Studies; ICD, International Classification of Diseases; ATC, Anatomical Therapeutic Chemical.

Table S2. Baseline Characteristics of Eligible Individuals Non-users of Antidepressants and/or Opioids from an Observational Emulation of a Target Trial

	**Aromatase inhibitors (n = 4,605)**	**Tamoxifen (n = 2,758)**
**Characteristics**	**n (%)**	**n (%)**
Age (years)[a]	68.0 [63.0, 75.0]	67.0 [61.0, 73.0]
Year of baseline		
2009	333 (7.2)	363 (13.2)
2010	501 (10.9)	442 (16.0)
2011	571 (12.4)	438 (15.9)
2012	599 (13.0)	403 (14.6)
2013	721 (15.7)	421 (15.3)
2014	941 (20.4)	351 (12.7)
2015	939 (20.4)	340 (12.3)
Time from diagnosis (days)	97.0 [59.0, 202.0]	67.0 [50.0, 101.8]
Stage at diagnosis		
Stage 1	2,404 (52.2)	2,100 (76.1)
Stage 2	2,024 (44.0)	642 (23.3)
Stage 3	177 (3.8)	16 (0.6)
T classification		
T1	2,404 (52.2)	2,100 (76.1)
T2	2,024 (44.0)	642 (23.3)
T3/T4	177 (3.8)	16 (0.6)
N Classification		
N0	3,915 (85.0)	2.672 (96.9)
N1/N2/N3	690 (15.0)	86 (3.1)
Grade		
Grade 1	638 (13.9)	812 (29.4)
Grade 2	2,454 (53.3)	1,690 (61.3)
Grade 3	1,307 (28.4)	230 (9.3)
Missing	206 (4.5)	26 (0.9)
HER2 status		
Positive	533 (11.6)	79 (2.9)
Negative	3,892 (84.5)	2,509 (91.0)
Missing	180 (3.9)	170 (6.2)
Side		
Right	2,190 (47.6)	1,385 (50.2)
Left	2,415 (52.4)	1,373 (49.8)
Chemotherapy	1,371 (29.8)	130 (4.7)
Radiotherapy	1,129 (24.5)	556 (20.2)
Antibody treatment	389 (8.4)	26 (0.9)
Cerebrovascular disease	93 (2.0)	23 (0.8)
Diabetes	289 (6.3)	123 (4.5)
COPD	87 (1.9)	46 (1.7)
Cardiovascular disease	1584 (34.4)	793 (28.8)
Diabetes drugs	295 (6.4)	121 (4.4)
Anticoagulants	1,242 (27.0)	466 (16.9)
NSAIDs	1,435 (31.2)	880 (31.9)
Hormone replacement therapy	1,267 (27.5)	839 (30.4)
Marital status		
Single	584 (12.7)	339 (12.3)
Married/cohabiting	2,783 (60.4)	1,711 (62.0)
Divorced/separated/widowed	1,238 (26.9)	708 (25.7)
Education status		
Pre-secondary education	1,282 (27.8)	708 (25.7)
High school education	1,899 (41.2)	1,126 (40.8)
Post-secondary education	1,372 (29.8)	898 (32.6)
Missing	52 (1.1)	26 (0.9)
Employment status		
Employed	2,662 (57.8)	1,675 (60.7)
Not employed	1,926 (41.8)	1,079 (39.1)
Missing	17 (0.4)	4 (0.1)

Note: Only grade, HER2 status, education status and employment status had missing data. Abbreviations: COPD, chronic obstructive pulmonary disease; NSAIDS, nonsteroidal anti-inflammatory drugs.[a] Values are expressed as median (interquartile range).

Table S3. Absolute Risks, Risk Differences, Risk Ratios and Hazard Ratios from the Observational Emulation of a Target Trial of Aromatase Inhibitors versus Tamoxifen on Death at 5 years, Sensitivity Analyses

	Aromatase inhibitors	Tamoxifen	RD [95% CI]	RR [95% CI]	HR [95% CI]
	Risk, % [95% CI]	Risk, % [95% CI]			
Sensitivity analyses					
ITT standardization [a]	12.4 [11.2–13.8]	9.0 [7.8–10.2]	3.4 [2.0–5.0]	1.37 [1.20–1.59]	1.22 [1.03–1.46]
Per-protocol standardization [b]	4.5 [3.8–5.6]	3.3 [2.6–4.0]	1.2 [2.0–2.4]	1.38 [1.06–1.82]	1.17 [0.90–1.54]
Complete case analysis	12.6 [11.4–13.9]	10.0 [8.4–11.8]	2.6 [0.5–4.5]	1.27 [1.04–1.53]	1.10 [0.90–1.35]

Note: ITT analysis was conducted using IPTW of baseline covariates.
Abbreviations: CI, confidence interval; RD, risk difference; RR, risk ratio; HR, hazard ratio; BIG 1-98, Breast International Group 1-98; ITT, intention-to-treat; IPTW, inverse probability treatment weighting; NR, not reported.
[a] Adjusted at baseline for: age, year of baseline, time from diagnosis, type of surgery, stage at diagnosis, T classification, N classification, grade, side, chemotherapy, radiotherapy, antibody treatment, cerebrovascular disease, diabetes, COPD, cardiovascular disease, diabetes drugs, anticoagulants, antidepressants, NSAIDs, opioids, hormone replacement therapy, marital status, education status and employment status.
[b] The model is adjusted for baseline and time-varying covariates.

Figure S1. Cumulative Incidence Curve from an Intention-to-Treat Analysis Observational Emulation of a Target Trial of Aromatase Inhibitors versus Tamoxifen on Death at 5 Years, within Non-users of Opioids and/or Antidepressants

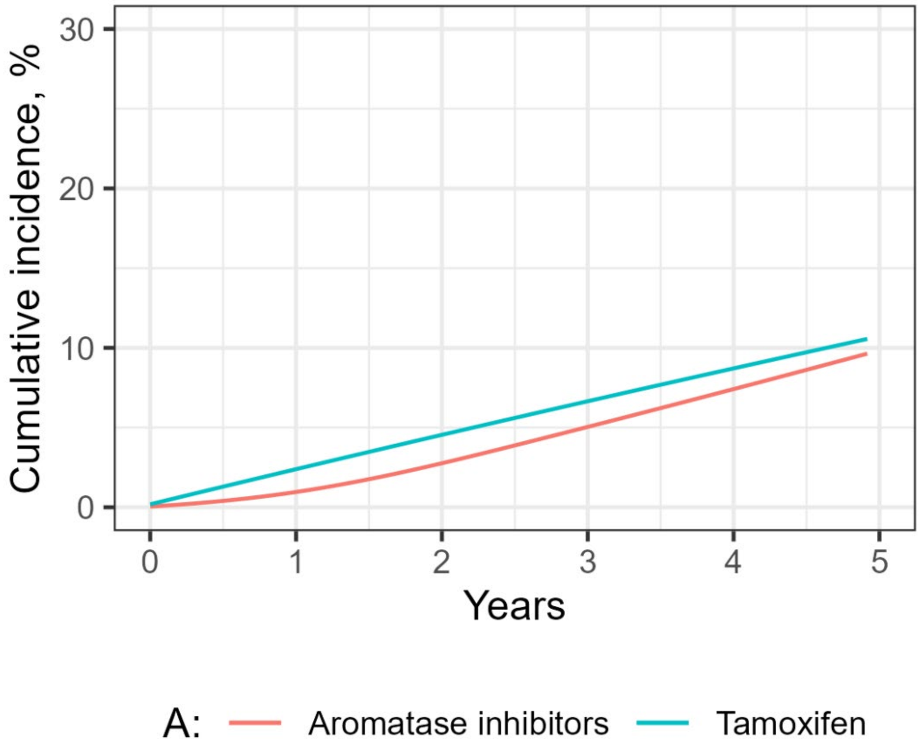

Supplement: Web_Material_kwaf183 [file web_material_kwaf183.zip › Supplementary material.pdf]
